# Unloading shoes for osteoarthritis of the knee: protocol for the SHARK randomised controlled trial

**DOI:** 10.1186/1471-2474-15-48

**Published:** 2014-02-21

**Authors:** Rana S Hinman, Tim V Wrigley, Ben R Metcalf, David J Hunter, Penny Campbell, Kade Paterson, Margaret P Staples, Kim L Bennell

**Affiliations:** 1 Centre for Health, Exercise and Sports Medicine, Department of Physiotherapy, School of Health Sciences, Faculty of Medicine Dentistry & Health Sciences, The University of Melbourne, Melbourne, VIC, Australia; 2 Royal North Shore Hospital, Rheumatology Department, and Kolling Institute, University of Sydney, Sydney, NSW, Australia; 3 Cabrini Institute and Monash University Department of Clincal Epidemiology at Cabrini, Malvern, VIC, Australia

## Abstract

**Background:**

Knee osteoarthritis (OA) is a common and disabling condition. Abnormalities in knee loading play an important role in disease pathogenesis, yet there are few non-surgical treatments for knee OA capable of reducing knee load. This two-arm randomised controlled trial is investigating the efficacy of specially-designed unloading shoes for the treatment of symptoms in people with knee OA.

**Methods/Design:**

164 people with symptomatic medial tibiofemoral joint OA will be recruited from the community and randomly allocated to receive either unloading shoes or control shoes. Unloading shoes have a specially-designed triple-density midsole where the medial side is softer than normal and the lateral side harder as well as a lateral wedge between the sole and sock-liner. Control shoes are standard athletic shoes and do not contain these features. Participants will be blinded to shoe allocation and will be instructed to wear the shoes as much as possible every day for 6 months, for a minimum of 4 hours per day. The primary outcomes are knee pain (numerical rating scale) and self-reported physical function (Western Ontario and McMaster Universities Osteoarthritis Index) measured at baseline and 6 months. Secondary outcomes include additional measures of knee pain, knee stiffness, participant global ratings of change in symptoms, quality-of-life and physical activity.

**Conclusions:**

The findings from this study will help determine whether specially-designed unloading shoes are efficacious in the management of knee OA.

**Trial registration:**

Australian New Zealand Clinical Trials Registry reference: ACTRN12613000851763.

## Background

Osteoarthritis (OA) is a leading cause of musculoskeletal pain and disability, particularly amongst older adults. In 2007, 20-25% of Australians over 55 years had OA [1]. This will further increase in coming years due to population ageing and rising obesity rates. Knee OA results in significant knee pain and physical dysfunction that typically worsen over time [2,3]. There is no cure, thus interventions that alleviate symptoms and prevent clinical decline over the long-term are required.

Knee OA results from increased joint loading acting within the context of systemic and/or local susceptibility [4]. As *in vivo* measurement of joint loads is not feasible, three-dimensional gait analysis is used to infer dynamic load. During walking, high loads are applied to the knee. An external knee adduction moment (KAM) is generated because the ground reaction force vector passes medially to the knee during stance [5]. This moment (or torque) tends to force the knee outwards, compressing the medial joint compartment and stretching lateral joint structures. In fact, abnormal medial load resulting from the KAM probably explains why knee OA occurs mostly in the medial tibiofemoral joint [6].

The KAM (measured as peak and/or the related KAM impulse) is a valid surrogate for medial knee load. The KAM is higher in people with medial knee OA [7-9] compared to controls, and it strongly predicts OA radiographic severity [10]. The KAM has also been shown to predict treatment outcome in patients undergoing high tibial osteotomy [11]. A higher KAM is associated with development of knee pain in older people [12], as well as with increased pain severity and physical dysfunction in people with knee OA [13,14]. Relationships between both the peak KAM and the KAM impulse and presence of medial knee bone marrow lesions have been demonstrated [15], which are an important source of pain in knee OA [16]. The KAM is one of only a few factors known to predict OA disease progression. A 1-unit increase in the peak KAM is associated with up to a 6.5-fold increase in the risk of disease progression over 6 years [13]. Thus, given its relationship to symptoms and clinical outcome in knee OA, there is increasing interest in mechanical interventions directed at lowering the peak KAM.

Clinical guidelines emphasise that appropriate footwear should be worn by people with knee OA [17]. This recommendation is based on expert opinion given the scant research evidence available. Wearing shoes significantly *increases* medial knee load compared to barefoot walking [18,19]. Recent research, by ourselves and others, has developed specialised shoes for people with knee OA with variable-stiffness soles that are denser laterally compared to medially in order to reduce the KAM [20-22]. Thus, when weightbearing, the greater medial compressibility effectively creates a laterally angled shoe that reduces the frontal plane lever arm of the ground reaction force at the knee joint centre, reducing the KAM [23,24]. These types of modified shoes can immediately reduce the KAM by 3.5-7.9% in people with medial knee OA [20,25]. In a single case study involving a patient with an instrumented knee replacement, variable-stiffness shoes reduced the peak KAM by 13.3% and medial joint contact force by 12.3% [22]. Furthermore, change in KAM correlated with change in contact force, validating the load-modifying effects of unloading shoes for knee OA.

Relative to their biomechanical effects, there is very little known about whether unloading shoes can reduce the pain and physical dysfunction associated with knee OA. There is only one randomized controlled trial (RCT) evaluating clinical benefits of variable-stiffness unloading shoes for knee OA [25,26]. The unloading shoes resulted in significant within-group pain reductions after 6 and 12 months of use, however the change in symptoms was not significantly different to that seen in the group wearing control shoes. This may be because of the relatively small sample size utilized (n = 79) and/or the large drop-out rate in the control group (n = 13 out of 39, 33%) by 6 months. Further RCTs are required before the efficacy of unloading shoes for managing the symptoms of OA can be known.

The primary aim of this study is to evaluate the efficacy of specialized unloading shoes on OA-associated symptoms in people with knee OA. We hypothesise that unloading shoes will reduce pain and improve self-reported physical function after 6 months when compared to control shoes.

## Methods/design

### Trial design

**Figure 1:**
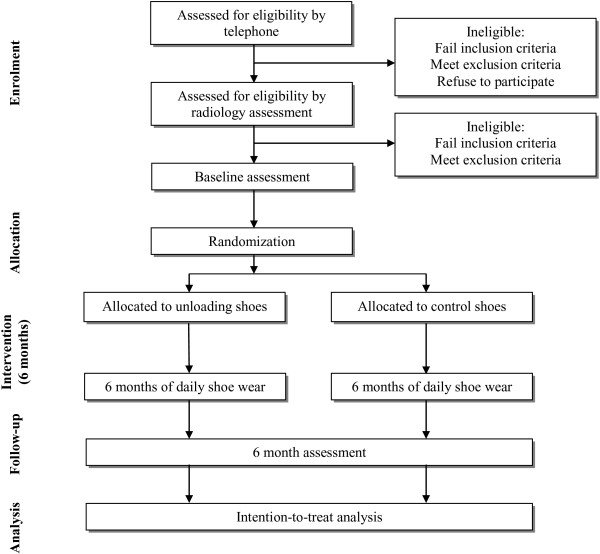
Flow diagram of study protocol.

The SHARK (SHoes for ARthritis of the Knee) trial is a two-arm participant- and assessor-blinded parallel-group RCT comparing unloading shoes to control (non-unloading) shoes (Figure 1). The protocol conforms to CONSORT guidelines for reporting of non-pharmacological interventions [27]. The primary end-point for analysis of outcomes is after 6 months of treatment.

### Participants

We will recruit 164 participants with medial tibiofemoral OA from the community via advertisements, media campaigns, social media (eg Facebook) and from our research volunteer database. Knee OA will be classified according to the American College of Rheumatology criteria [28]. Participants will be included if they i) are aged ≥50 years ii) report knee pain on most days of the past month; iii) report a minimum average pain score of 4 on an 11-point numerical rating scale (NRS, with terminal descriptors of ‘no pain’ and ‘worst pain possible’) in the past week; iv) demonstrate definite x-ray evidence of OA (Kellgren & Lawrence [29] Grade ≥ 2) and; v) demonstrate definite medial tibiofemoral compartment OA on x-ray (defined as ≥ grade 1 medial osteophytes AND ≥ grade 1 medial joint space narrowing that is greater than lateral joint space narrowing, using a standard atlas [30]).

Participants will be excluded if they i) have more severe lateral tibiofemoral compartment osteophytes relative to medial; ii) have undergone intra-articular corticosteroid injection or knee surgery to either knee within past 3 months; iii) have a systemic arthritic condition; iv) have had a knee joint replacement or high tibial osteotomy in the past, or plan to undergo surgery to either knee in next 6 months; v) have any other muscular, joint or neurological condition affecting lower limb function; vi) current or previous (within 6 months) use of shoe inserts, knee or ankle braces or customized shoes prescribed by a health professional; vii) are unable to walk unaided; viii) have a body mass index ≥ 36 kg/m^2^ (due to difficulties in three-dimensional gait analysis) or; ix) have ankle/foot pathology/pain on either side.

### Procedure

Figure 1 outlines the trial phases. Volunteers will undergo initial screening via an online survey. Further screening will then occur via questioning over the telephone. Potentially eligible volunteers will then undergo x-ray screening to confirm eligibility. Baseline and 6-month assessments (primary time-point) will be carried out at the Department of Physiotherapy, the University of Melbourne. Additionally, participants will complete monthly log-books at home (mailed back to the researchers), will monitor daily physical activity levels for one week during months 2 and 5, and will complete primary outcome measures at home (mailed back to researchers) at 3 months (secondary time-point). Ethical approval has been obtained from the University of Melbourne Human Research Ethics Committee (HREC No. 1239045). All participants will provide written informed consent. For participants with bilaterally eligible knees, only the most symptomatic knee (as identified by the participant) will be evaluated, although the allocated shoes will be worn bilaterally.

### Randomisation and allocation concealment

Eligible participants will be randomised following baseline data collection of primary and secondary outcome measures to receive either unloading shoes or control shoes. Randomisation will be by random permuted blocks of size from 6 to 12 and stratified by radiographic disease severity (Kellgren and Lawrence grades 2, 3 and 4 [29]). The randomisation schedule will be prepared by the study biostatistician. To conceal randomisation, consecutively numbered, sealed, opaque envelopes will be used and kept in a locked location.

Participants will be blinded to which treatment group they are allocated and allocated shoes will have no identifying model or brand. The unloading shoes are visually similar to the control shoes to maintain participant blinding. Participants will be asked to indicate which group they believe they are in order to measure the success of blinding. As all primary and secondary outcomes are participant-reported questionnaires, and participants will be blinded to group allocation, the assessor is also blinded in this study. An investigator, who will also be blinded to group allocation, will oversee administration of all primary and secondary outcome measures. Additional biomechanical and gait analysis measures (that will not be used to determine treatment efficacy but will be used in subsequent analyses to evaluate predictors of treatment response) will be conducted by another investigator who is not blinded to group allocation by necessity due to the nature of the biomechanical testing procedures.

### Interventions

Participants will be provided with a pair of shoes (allocation according to the randomisation schedule) appropriately sized to their feet. Participants will be instructed to wear their allocated shoes as much as possible every day for 6 months, and to avoid wearing their usual shoes as much as possible. At a minimum, participants will be asked to wear the shoes for at least 4 hours every day. Participants will be advised to initially wear the shoes for one hour, and thereafter increase by one hour each day until wearing the shoes for all waking hours.

### Unloading shoes

Black recreational walking shoes (Gel-Melbourne OA, Asics) that have been designed to unload the medial compartment of the knee [20] will be used. These shoes have a specially-designed triple-density sole of compression moulded ethylene vinyl acetate, where the lateral midsole is stiffer than the medial. They also contain a thin, full-length, half-width laterally-biased wedge (approximately 5 degrees angulation) attached to the underside of the sock-liner.

#### Control shoes

Black neutral recreational walking shoes (Gel-Odyssey, Asics) that are similar in appearance to the unloading shoes, but do not contain the the stiffer lateral midsole nor a lateral wedge insert will be used.

### Treatment adherence

Treatment adherence will be self-reported in a log-book. For the fourth week of each month (to reduce the burden on participants), participants will record how many hours each day they wear their allocated shoes, for 7 consecutive days. They will also rate their perceived overall level of compliance over the past month with the instruction to wear their allocated shoes for a minimum of 4 hours per day on an 11-point NRS (with terminal descriptors of ‘not at all’ and ‘completely as instructed’). Log-books will be posted back to investigators on a monthly basis. Participants will also rate their overall level of compliance over the 6-month treatment period using an identical NRS at the 6-month assessment. At this time, participants will also be asked to estimate, on average, how many hours per day they wore their allocated shoes for the 6-month treatment period.

Treatment adherence (over a one-week interval) will further be monitored at two time-points (2 months and 5 months) by the use of pedometers. For 7 consecutive days, participants will be asked to attach and wear a shoe pedometer (Oregon Scientific PE903, Oregon, USA) to one of their allocated trial shoes. Participants will also be asked to wear a second pedometer at their waist during waking hours (Omron HJ720ITC Pedometer, Omron Healthcare, Illinois, USA) over the same period in order to capture daily step counts irrespective of footwear worn. For each participant at each time-point, we will calculate the proportion of daily steps taken whilst wearing their allocated shoes. Participants will be telephoned prior to the week of pedometer data collection to facilitate adherence with pedometers.

### Adverse effects of treatment, shoe comfort and co-interventions

Adverse events will be defined as any problem believed by participants to be caused by the allocated shoes that lasted for two days or more and/or caused the participant to take medications or see a health professional. Participants will record any adverse effects of treatment in the monthly log-books. At the 6-month assessment, adverse effects will also be ascertained by open-ended questioning by the blinded assessor.

Monthly log-books will also be used to record participant ratings of shoe comfort. Participants will rate their overall level of shoe comfort experienced over the past month via an 11-point NRS (with terminal descriptors of ‘extremely uncomfortable’ and ‘extremely comfortable’). Participants will also rate their overall level of shoe comfort over the 6-month treatment period using an identical NRS at the 6-month assessment.

Use of co-interventions (medications for knee pain and any other treatments for knee OA) will also be recorded in the monthly log-books. At the 6-month assessment, use of co-interventions will also be ascertained by open-ended questioning by the blinded assessor.

### Outcome measures

Table 1 summarises the outcome measures that are being collected to determine treatment efficacy. Our primary outcomes are recommended validated measures of pain and physical function for knee OA [31]. These will be measured at baseline, 3 months and 6 months. Conclusions regarding treatment efficacy will be based on the changes in primary outcome measures from baseline to 6 months. Our two primary outcomes are:

**Table 1:** Summary of primary and secondary outcome measures collected to determine treatment effectiveness

**Outcome**	**Measurement tool**
**Primary**	
Knee pain on walking	11-point numerical rating scale
Physical function	WOMAC physical function subscale
**Secondary**	
Global rating of change in:	7-point scales
i) Knee pain	
ii) Physical function	
Knee pain	WOMAC pain subscale
Knee pain	ICOAP
Knee stiffness	WOMAC stiffness subscale
Health-related quality of life	AQoL-6D
Physical activity	PASE

WOMAC = Western Ontario and McMaster Universities Osteoarthritis Index; PASE = physical activity scale for the elderly; ICOAP = Intermittent and Constant Osteoarthritis Pain questionnaire; AQoL-6D = Assessment of Quality of Life instrument (version 6D).

#### Knee pain on walking measured by an 11-point NRS

Overall average pain on walking over the past week will be self-reported via a NRS with terminal descriptors of ‘no pain’ (score 0) and ‘worst pain possible’ (score 10). Such measurement has demonstrated reliability in OA [32].

#### Physical function measured by the function subscale of the WOMAC

Difficulty with physical functioning will be measured by the Western Ontario and McMaster Universities (WOMAC) Osteoarthritis Index (Likert version 3.1) [33]. This is a disease-specific self-report instrument whose validity, reliability and responsiveness have been demonstrated in an extensive range of OA studies [34]. The physical function subscale contains 17 questions (each answered on a Likert scale where 0 = no dysfunction and 4 = extreme dysfunction) and has a total score ranging from 0 (no dysfunction) to 68 (maximum dysfunction).

Our secondary outcome measures, which will be administered at baseline and at 6 months (with the exception of some outcomes as detailed below), include:

#### Participant-perceived response to treatment measured on 7-point scales

Participants will rate their overall global change in a) pain and b) physical function since enrolling in the study, at 3 months and 6 months. The terminal descriptors on the 7-point scales will be ‘much worse’ to ‘much better’ [35]. Participants who report ‘moderately better’ or ‘much better’ will be classified as improved.

#### Knee pain measured by the WOMAC pain subscale

This subscale contains 8 questions (each answered on a Likert scale where 0 = no pain and 4 = extreme pain) and has a total score ranging from 0 (no pain) to 20 (maximum pain). In addition to baseline and 6 months, this will also be administered at 3 months.

#### Knee stiffness measured by the WOMAC stiffness subscale

This subscale contains 2 questions (each answered on a Likert scale where 0 = no stiffness and 4 = extreme stiffness) and has a total score ranging from 0 (no stiffness) to 8 (maximum stiffness). In addition to baseline and 6 months, this will also be administered at 3 months.

#### Knee pain measured by the ICOAP

The Intermittent and Constant Osteoarthritis Pain (ICOAP) questionnaire [36] will also be used to assess knee pain. The ICOAP contains 11 questions, each scored from 0–4, giving a range of possible scores from 0 (no pain) to 44 (maximum pain).

#### Health-related quality of life measured by the AQoL-6D

The Assessment of Quality of Life (AQoL) instrument [37] (version AQoL-6D) contains 20 items assessing independent living, mental health, relationships, pain, coping and senses. Scores range from -0.04 to 1.00 and higher scores indicate better quality of life.

#### Physical activity levels measured by the PASE

The Physical Activity Scale for the Elderly (PASE) measures physical activity over the previous week [38]. Scores range from 0 to over 400, with higher scores indicating greater physical activity.

### Additional measures

A range of additional measures will be collected for the purposes of answering related questions about biomechanical effects of the unloading and control shoes, and for subsequent analyses of potential mediating effects on clinical outcomes. These measures will not be used, however, to determine treatment efficacy. Additional measures that will be obtained include radiographic measures of knee alignment; the painDETECT questionnaire for identifying neuropathic pain [39]; a rating of participant expectation of treatment outcome measured on a 5-point ordinal scale; objective measures of foot posture, mobility and anthropometry including the Foot Posture Index [40]; and lower limb kinetics and kinematics during walking measured with three-dimensional gait analysis (Vicon, Oxford, UK; AMTI, Massachusetts, USA) and; in-shoe regional foot pressure patterns (Novel Pedar, Munich, Germany).

### Sample size calculations

The trial is powered to detect clinically important changes in the primary outcomes of knee pain on walking (NRS) and change in physical function (WOMAC). The minimum clinically important difference to be detected in OA trials is a change in pain of 1.8 units (out of 10) [41] and change in function of six units (out of 68) [42]. Our calculations are based on SD’s from our unpublished pilot data of the effects of unloading shoes, and on control group data from our previous RCT of lateral wedged insoles [43]. We assume a between-subject SD of 2.5 for pain and 10.5 units for WOMAC physical function, and a baseline to 12-month correlation in scores of 0.29 for pain and 0.51 for physical function. These assumptions, together with an analysis of covariance adjusted for baseline scores, require a sample size of 41 participants per group to achieve 90% power to detect the minimum clinically important difference in pain. A similar analysis for function scores requires 65 participants per group. Allowing for a 20% attrition rate, we will recruit 82 participants per arm, or 164 participants in total.

### Statistical analyses

Our biostatistician will analyse data in a blinded manner, with p values less than 0.05 considered significant. Main comparative analyses between groups will be performed using intention-to-treat. This analysis will include all participants including those who have missing data and those who do not fully adhere to the protocol. To account for missing data, multiple imputation of missing follow-up measures, assuming data are missing at random and follow a multivariate normal distribution, will be performed as a sensitivity analysis.

Demographic characteristics, as well as baseline scores on primary and secondary outcome measures, will be presented to assess baseline comparability of treatment groups. These variables will also be examined for those participants who withdraw from the study and those who remain.

Descriptive statistics will be presented for each group as the mean change (standard deviation, 95% confidence intervals) in the outcomes from baseline to each time-point. For continuous outcome measures, differences in mean change (baseline minus follow-up score) will be compared between groups using linear regression random effects modelling adjusted for baseline values of the outcome. Model diagnostic checks will utilise residual plots. Similar regression models for binary and ordinal outcome measures will use random effects logistic and proportional odds models, respectively. We will also perform a per protocol analysis as appropriate.

### Timelines

The application for project funding was successful in October 2012 and funding commenced in June 2013. Ethics approval was obtained from the Human Research Ethics Committee of the University of Melbourne in February 2013. Recruitment commenced in August 2013 will be completed in December 2015. The trial is due for completion in June 2016 when all participants will have completed 6-month follow-up.

## Discussion

This paper has presented the theoretical rationale, as well as the protocol, for a RCT that is testing the efficacy of specially-designed unloading shoes for managing the symptoms of knee OA. The findings of this study will help determine whether unloading shoes can relieve pain and/or improve self-reported physical function over 6 months of use. Given that clinical guidelines emphasise appropriate footwear should be worn by people with knee OA [17], and there is scant evidence from clinical trials to inform optimal footwear choices in this population, findings from the current RCT will assist clinicians and people with knee OA in selection of appropriate footwear to manage symptoms of OA.

## Competing interests

RSH, TVW and KLB, and the University of Melbourne, receive royalties from sales of the unloading shoes that are being tested in this study. The manufacturer of the unloading shoes played no role in the study design nor will have any input into the analysis and interpretation of data from this study.

## Authors’ contributions

RSH, TVW and KLB conceived the project and RSH is leading the trial. RSH, TVW and DJH and KLB developed the protocol and procured the project funding. TVW devised the biomechanical data collection procedures. PC recruits and screens the participants, administers the primary and secondary measures and performs data entry. BM randomises participants to groups, fits the allocated shoes and conducts biomechanical assessments. KP oversees the assessment of foot posture and foot function and associated biomechanical measures. MPS performed the sample size calculations and designed the statistical analyses. RSH wrote the first and final draft of the manuscript. All authors participated in the trial design, provided feedback on drafts of this paper and read and approved the final manuscript.

## Pre-publication history

The pre-publication history for this paper can be accessed here:

http://www.biomedcentral.com/1471-2474/15/48/prepub
